# Presumed post-mortem donors: the degree of information among university students

**DOI:** 10.1186/s12910-021-00707-2

**Published:** 2021-10-16

**Authors:** Rita da Silva Clemente Pinho, Cristina Maria Nogueira da Costa Santos, Ivone Maria Resende Figueiredo Duarte

**Affiliations:** 1 Faculty of Medicine, University of Porto, Al. Prof. Hernâni Monteiro, 4200-319 Porto, Portugal; 2 Department of Community Medicine, Information and Health Decision Sciences, Faculty of Medicine, University of Porto - Centre for Research in Health Technologies and Information Systems (CINTESIS), Porto, Portugal

**Keywords:** Organ transplantation, Post-mortem donor, Autonomy, Opt-out system, Presumed consent

## Abstract

**Background:**

Organ transplantation represents the most effective and acceptable therapy for end-stage organ failure. However, its frequent practice often leads to a shortage of organs worldwide. To solve this dilemma, some countries, such as Portugal, have switched from an opt-in to an opt-out system, which has raised concerns about respect for individual autonomy. We aimed to evaluate whether young university students are aware of this opt-out system so that they can make informed, autonomous and conscious decisions, as well as to identify the factors that determine a positive attitude toward post-mortem organ donation.

**Methods:**

An observational, cross-sectional study was developed and a questionnaire was administered to first-year students from six faculties of the University of Porto.

**Results:**

Of the 841 participants, 60% were unaware that Portugal had adopted an opt-out system. Among the informed individuals, their main sources of information included social media, internet, and family. Furthermore, only 48% of all participants agreed with the current opt-out system. Female sex (*p* = 0.049; OR 1.393), knowledge of the law (*p* < 0.001; OR 4.749) and family being the primary source of information (*p* < 0.001; OR 2.855) were independent factors associated with a positive attitude toward post-mortem organ donation law.

**Conclusions:**

There is a significant lack of knowledge among young university students regarding the presumed post-mortem organ donation law and how it works. Female sex, having family as a primary source of information and being aware of the presumed post-mortem organ donation law are the strongest independent factors that determine a positive attitude toward the opt-out system.

## Background

Organ transplantation is the most effective and acceptable therapy for end-stage organ failure [1–5]. The first successful organ transplant was a kidney transplant performed by Dr. Joseph Murray in 1954, who received the 1990 Nobel Prize in Medicine for his work [6]. The first transplant in Portugal was a kidney transplant from a living donor performed by Professor Linhares Furtado in 1969 [7], while the first transplant from a deceased donor was also a kidney transplant, performed in 1980 [8]. From there, various types of transplants were performed, including the first heart transplant in 1986 and the first hepatic and pancreatic transplants in 1988 and 1993, respectively [8].

However, a gap between organ needs and supply exists [4, 9], which has led to a consistent shortage of organs [3, 4, 10–15]. This represents the biggest obstacle to increasing the number of organ transplantations performed [1, 4] and has led to an increased number of patients on the transplant waiting list [1, 16]. Consequently, most patients on these lists die before surgery is performed [1, 16, 17], although this is not true for all transplants. Patients with kidney failure have other treatment options, such as dialysis and living donor transplant, while those with hepatic failure may receive a transplant from a living donor due to the great regenerative capacity of the liver.

In an attempt to close the organ shortage gap, some countries, such as Austria, France, Greece, Poland, Spain, Sweden, Wales and, most recently, England, have adopted an opt-out system [18]. The basis of this system is presumed consent, which is invoked when the minimum conditions for obtaining express consent are not met and when there are no objective and reliable data to suggest that the patient would oppose a particular intervention [19]. Thus, in this system, individuals are presumed to be willing donors after death and are considered to have consented to organ donation unless they registered as non-donors in life [4]. The opt-out system has two main subtypes: a ‘hard opt-out system’, where family members do not have the right to refuse post-mortem organ recovery, and a ‘soft opt-out system', where the will of the family is considered. Under the latter system, if the family rejects organ recovery, organs cannot be removed from the deceased person [4].

On the one hand, in a systematic review, Rithalia et al. [20] find a positive association between presumed consent laws and organ donation rates, although they conclude that presumed consent alone could not explain the variation in organ donation rates between different countries. Indeed, there are other important factors associated with this variation, such as a country’s traffic accident mortality rates, transplantation capacity, health expenditure per capita and gross domestic product [20]. On the other hand, a cross-sectional study by Arshad et al. [21] finds no significant rise in organ donation rates after switching from an opt-in to an opt-out system.

Portugal switched from an opt-in to an opt-out system in the 1900s. Until 1993, an individual who wanted to be a donor had to communicate this to the Ministry of Health by express consent. In 1993, Portuguese law was changed to allow for presumed consent under Article no. 10 of Law no. 12/93 of 22 April, which states that “all national citizens and stateless persons and foreigners living in Portugal who have not reported their non-donor status to the Ministry of Health are considered potential post-mortem donors” [22]. With regard to minors or people with disabilities, their non-donor status must manifested by their legal representatives or may be expressed by minors who have demonstrated their capacity to understand and express their unwillingness to donate. Indeed, Portuguese law states that, regarding matters of health, informed consent is valid only if it is provided by those who are over 16 years old and have enough judgement to assess its meaning and scope at the time it is being provided [23]. Simultaneously, the computerized National Register of Non-Donors (RENNDA) was created to record all individuals who have expressed their non-donor status to the Ministry of Health. People registered in RENNDA also receive a non-donor card.

Portugal has become one of the countries with the highest organ donation rate per million inhabitants; in 2009, it was considered the second-highest European country in terms of donation rate [7]. The latest data published by the Portuguese Institute of Blood and Transplantation (IPST) show that, between 2016 and 2018, the number of post-mortem donors remained constant, but was higher than in 2011 [24].

When the opt-out system was implemented, Portuguese transplantation law provided for post-mortem donations only after the confirmation of brain death [22]. Indeed, another measure that could be used to increase the donation rate would be to extend the concept of post-mortem donors to individuals declared dead according to cardiorespiratory criteria [25, 26]. In Portugal, organ donation after circulatory death is only allowed for individuals in Category II of the Maastricht classification—individuals deceased outside or inside the hospital who were subjected to timely and adequate resuscitation maneuvers that were ultimately unsuccessful; after individuals are declared dead, they are maintained on life support to preserve the viability of organs for donation [8]. To this end, Portuguese law was updated and Dispatch no. 14341/2013 of 6 November [27] now includes cardiorespiratory and brain death criteria to verify death for donation purposes. The first organ transplant involving an individual declared dead according to cardiorespiratory criteria was performed on 1 January 2016 at the São João University Hospital in Porto.

One of the biggest issues in the discussion regarding presumed post-mortem organ donation is the ability of individuals to exercise their autonomy. According to Beauchamp and Childress, the autonomous individual acts freely in accordance with a self-chosen plan, and to respect individual autonomy is necessarily to acknowledge that individuals have the right to hold views, make choices and act in accordance with their values and beliefs [28]. Mackay and Robinson also defend that autonomy refers to each individual’s ability to decide what to do with their bodies on the basis of their values [29].

Indeed, respect for autonomy requires that healthcare professionals act in accordance with the wishes of the individual while alive [30], whether opt-out systems respect this individual autonomy is a topic of debate. On the one hand, according to MacKay and Robinson [29, 30], having an opt-out system in place among a poorly informed population can be viewed as coercive and disrespectful of individual autonomy. On the other hand, Saunders [31] considers the opt-out system to preserve individual freedom of choice, assuming that people are informed and that, therefore, those who do not opt out are consenting to the use of their organs. Other authors argue that governments have the responsibility to defend individual autonomy [7, 30]. Pereira [7], for example, concludes that an opt-out system would truly respect individual autonomy only if the government were to invest in public information. Similarly, Mackay [30] claims that, if they are to respect individual autonomy, governments must take action to inform the population about the law, system and possibility of registering as non-donors, thereby preventing organ collection from those who do not consent.

In this sense, in order to respect the principle of autonomy, the population must be informed about the opt-out system. Nevertheless, in their systematic review, Molina-Pérez et al. [18] conclude that people in opt-out countries are poorly informed about the system of presumed post-mortem organ donation. Furthermore, according to Rithalia et al. [20], the rate of organ donation is higher in opt-out countries because people are less knowledgeable about the law. This is contrary to the law in opt-out countries since, according to these policies, the government has a duty to inform the population by means of public campaigns [18]. Indeed, if an individual does not know about their status as a presumed post-mortem organ donor, their organs could be retrieved against their will. This possibility destroys the ethical basis of presumed consent since it annuls any respect for self-will, autonomy and human rights.

Portuguese law regarding post-mortem organ donation—Article no. 15 of Law no. 12/93 of 22 April [22]—states that the government should promote information campaigns regarding organ retrieval for transplantation as well as information campaigns clarifying the possibility of refusing post-mortem donation through registration in RENNDA. The aim of this would be to ensure that any Portuguese citizen, stateless person or foreigner residing in Portugal possesses all available information on this subject and would thus be able to make an informed, autonomous and conscious decision.

Only one study was found on the Portuguese population’s perception of the post-mortem organ donation law [32]. The author points out that, although most participants agreed with the opt-out system, they did not know that this was the system in place, were not aware of the legislation and did not have enough information to make an autonomous decision on whether or not to be organ donors.

Given that only one study on the case of Portugal was found and the international literature revealed a lack of information on the populations of opt-out countries, our study was conducted to evaluate whether Portuguese students enrolled in their first year of university education are aware of this opt-out system and to identify the factors that determine a positive attitude toward post-mortem organ donation.

## Methods

### Study population and sampling techniques

The selected population in this quantitative, observational study included first-year university students enrolled in six faculties of the University of Porto during the 2019/2020 academic year. Three of these faculties were related to social or health sciences: The Faculty of Medicine of the University of Porto (FMUP), the Faculty of Nursing of the University of Porto (ESEP), and the Faculty of Psychology and Education Sciences of the University of Porto (FPCEUP). The other three were not related to sciences: The Faculty of Law of the University of Porto (FDUP), the Faculty of Architecture of the University of Porto (FAUP), and the Faculty of Sports of the University of Porto (FADEUP).

These students constitute a strategic population stratum: they are at least 18 years old and therefore adults under Portuguese law who can exercise their civil autonomy; it is possible to evaluate whether information about the law and system of presumed post-mortem organ donation was transmitted during their education; only first-year students were admitted to the study to prevent new information on the topic (such as that which could be given during university studies) from affecting the results; they were selected from six distinct faculties of the University of Porto to assess whether their chosen course had any influence on the knowledge of the law they may have acquired in their individual education before entering university; finally, they constitute a heterogenous sample, in that some completed their secondary education in the public system while others completed it in the private system, and it could be expected that yet others may be from foreign countries.

### Instrument

An original questionnaire was designed (see “Appendix”). This questionnaire was subdivided into two sections, the first containing 7 sociodemographic questions and the second containing 17 items relating to: (1) knowledge about post-mortem organ donation law and how it works; (2) knowledge about the concept of brain death; (3) which of the available information resources were used and the quality of these resources; (4) participants’ attitudes toward post-mortem organ donation; and (5) whether or not the topic should be included in secondary school curricula.

A pilot survey was carried out in which the questionnaire was administered to 10 final-year medical students of both sexes to identify and exclude interpretation biases.

The study was conducted between October and November 2019. As previously agreed upon with the director of each faculty, the questionnaire was anonymously self-completed over a 5–10 min period during class under the supervision of the principal researcher.

### Statistical analysis

Answers to the questionnaire were described in absolute and relative frequencies. These frequencies were compared with the chi-square test or Fisher’s exact test. A logistic regression was performed to identify the factors that determine a positive attitude toward post-mortem organ donation. A multivariate logistic model was adjusted by stepwise forward conditional selection with significant variables associated with a positive attitude toward post-mortem organ donation.

A significance level of 0.05 was considered and the Statistical Package for Social Sciences (IBM-SPSS) was used to analyze the data.

## Results

### Demographic results

The response rate for each faculty was calculated considering the total number of students present in the class where the questionnaire was administered. Given that no student under these conditions refused to participate in the study, the response rate was 100% for each faculty and the questionnaire completion rate was 89%.

In terms of how students were distributed across the faculties, 163 were from FDUP, 155 were from FMUP, 145 were from ESEP, 144 were from FPCEUP, 124 were from FAUP and 110 were from FADEUP (average age 18.17 years old). There were differences between faculties regarding the percentage of women (*p* < 0.001), but the majority of participants were women in all faculties, with the exception of FADEUP. Differences between faculties were found regarding religious beliefs (*p* < 0.001), although the majority of students claimed to be Christian in all faculties except for FPCEUP, where only half of students practiced this religion. Approximately 98% (n = 821) of all respondents taking part in the survey were single. See Table 1.

**Table 1:** Description of the student sample

	Faculty						*p*
	ESEP	FADEUP	FAUP	FDUP	FMUP	FPCEUP	
	n (%)	N (%)	n (%)	N (%)	N (%)	N (%)	
*Sex*							**< 0.001**
Female	121 (83)	29 (26)	85 (69)	121 (74)	113 (73)	114 (79)	
Male	24 (17)	81 (74)	39 (31)	42 (26)	42 (27)	30 (21)	
*Marital status*							0.137
Single	139 (96)	107 (97)	123 (99)	162 (99)	151 (97)	139 (97)	
Married	4 (3)	3 (3)	0 (0)	1 (1)	3 (2)	1 (1)	
Widower/widow	0 (0)	0 (0)	1 (1)	0 (0)	0 (0)	0 (0)	
Divorced	0 (0)	0 (0)	0 (0)	0 (0)	0 (0)	1 (1)	
Common-law marriage	2 (1)	0 (0)	0 (0)	0 (0)	1 (1)	3 (2)	
*Religion*							**< 0.001**
None	27 (19)	32 (29)	52 (42)	67 (41)	46 (30)	63 (44)	
Christian	118 (81)	77 (70)	71 (57)	96 (59)	109 (70)	72 (50)	
Other	0 (0)	1 (1)	1 (1)	0 (0)	0 (0)	8 (6)	

A significance level (*p*) of 0.05 was considered (bolded values)

ESEP, Faculty of Nursing of the University of Porto; FADEUP, Faculty of Sports of the University of Porto; FAUP, Faculty of Architecture of the University of Porto; FDUP, Faculty of Law of the University of Porto; FMUP, Faculty of Medicine of the University of Porto; FPCEUP, Faculty of Psychology and Education Sciences of the University of Porto

About 90% (n = 753) of all participants completed secondary school in Portugal (the majority in the Northern region) and 27% (n = 202) of these studied in private schools; 3% (n = 25) of all participants finished their secondary education in African countries where the official spoken language is Portuguese (PALOP countries), while about 7% (n = 60) of participants finished their secondary education in non-African countries where Portuguese is the official spoken language (non-PALOP countries), mainly Brazil.

### Knowledge of post-mortem organ donation law and how it works

Differences between faculties were found in terms of awareness of current Portuguese legislation (*p* < 0.001) and awareness that organ sales are forbidden (*p* < 0.001). Of all students, 60% (n = 505) were unaware of current Portuguese legislation, since they did not know that all national citizens, stateless persons and foreigners living in Portugal who have not reported their non-donor status to the Ministry of Health are considered potential post-mortem donors; medicine and nursing students were most likely to be aware of the law. Most participants (79% [n = 666]) were aware that organ sales are forbidden, however, 28% (n = 35) of architecture students were unsure of this topic. Approximately 43% (n = 361) of respondents demonstrated knowledge regarding the legal framework applicable to minors and disabled people, with no differences between faculties.

Most often, the students who were aware of the law were the students who most agreed with it (*p* < 0.001).

Only 7% (n = 58) of all students stated that they knew how to register themselves as non-donors; of these, 2% (n = 13) were registered non-donors.

Only 51% (n = 430) of all respondents considered themselves to be presumed post-mortem donors. Nursing and medical students were most likely to be aware of this condition, while more women than men were knowledgeable about being a presumed post-mortem donor (56% versus 41%; *p* < 0.001). Portuguese students were more likely to be knowledgeable about being a presumed post-mortem donor than students who came from PALOP and non-PALOP countries (54% versus 27% and 22%, respectively; *p* < 0.001). No significant differences were found between awareness of the law and religious beliefs and between awareness of the law and the Portuguese education system (public versus private secondary schools).

### Knowledge on the concept of brain death

Of all students, 93% (n = 784) correctly understood the concept of brain death, correctly affirming that brain death occurs when the brain has no functionality, even when the heart continues to beat through artificial life support. However, only 64% (n = 538) were aware that individuals diagnosed with irreversible brain death but who continue to be sustained by artificial life support are potential post-mortem organ donors. Differences between faculties were found in the perception of brain death (*p* < 0.001), with medical students demonstrating a greater knowledge on the subject of brain death.

### Means of information used and the level of information quality

Among participants, 36% (n = 304) said that they *did not know* about post-mortem organ donation legislation. Approximately 27% (n = 229) of participants had received information from social media and 26% (n = 219) had received information from the internet in general. About 22% (n = 188) mentioned family as a primary source of information, while 10% (n = 82) said that they were informed by health professionals. Only 6% (n = 46) of participants had been informed by public campaigns. Portuguese students talked more about organ donation within their families than students who came from non-PALOP countries (24% versus 10%; *p* = 0.036). Consequently, 74% (n = 139) of participants who said that family was their primary source of information agreed with the law (*p* < 0.001). No significant differences were found between the secondary school education system in Portugal (private versus public) and the information resources used (*p* = 1.000).

Among all respondents, 62% (n = 524) considered the available information to be insufficient, whereas 14% classified it as being confusing, 12% as inexistent, and only 10% considered the available information to be sufficient.

About 3% (n = 19) of students who came from Portuguese secondary schools said that post-mortem organ donation was a subject that had been broached in classes. Approximately 21% (n = 12) of participants who came from non-PALOP countries had discussed the subject in secondary school (*p* < 0.001). Of all respondents, 88% (n = 737) believed that this subject should be talked about in school. No significant differences were found between the secondary school education system in Portugal (private versus public) and participants’ respective approaches to the topic.

### Students’ attitudes toward post-mortem organ donation

Only 48% (n = 404) of all students agreed with the law, and differences were found between faculties (*p* < 0.001): approximately 60% (n = 92) of medical students were in favor; 56% (n = 61) of FADEUP students, 54% (n = 66%) of FAUP students and 45% (n = 73) of FDUP students were undecided and nearly 10% (n = 16) of law students did not agree at all.

Women were more in favor of the legislation than men (52% versus 42%; *p* = 0.037).

Regarding students who considered themselves to be presumed post-mortem organ donors, 68% (n = 281) agreed with the law, 28% (n = 118) were undecided and 5% (n = 20) were against it. In terms of those who were unsure about being a presumed donor, only 29% (n = 116) were in favor, while 4% (n = 17) answered negatively (*p* < 0.001).

Of the students who were undecided about the possibility of organ donation from a brain-dead person, only 37% (n = 69) were in favor of the post-mortem organ donation law (*p* < 0.001).

No significant differences were established between religion and being in favour of the legislation (*p* = 0.865).

### Multivariate analysis of factors that determine attitude toward donation

Variables including female sex, knowledge of the post-mortem organ donation law, having family as the primary source of information, knowledge about the possibility of organ donation from a brain-dead person and being a medical student (FMUP) were significantly associated with a positive attitude toward post-mortem organ donation. Despite this, only the following variables persisted as independent factors after the odds ratios were adjusted (as seen in Table 2): female sex (*p* = 0.049; OR 1.393), knowledge of the law (*p* < 0.001; OR 4.749) and family as the primary source of information (*p* < 0.001; OR 2.855).

**Table 2:** Factors affecting attitude toward post-mortem organ donation: a multivariate analysis

	Crude		Adjusted	
	OR	*p*	OR	*p*
*Sex*				
Male	Ref		Ref	
Female	1.453	**0.014**	1.393	**0.049**
*Knowledge of the law*				
No/don’t know	Ref		Ref	
Yes	5.663	** < 0.001**	4.749	** < 0.001**
*Family as the primary source of information*				
No/don’t know	Ref		Ref	
Yes	4.001	** < 0.001**	2.855	** < 0.001**
*Knowledge about the possibility of organ donation from a brain-dead person*				
No/don’t know	Ref		–	
Yes	1.522	**0.004**	–	–
*Faculty*				
FMUP	Ref			
ESEP	0.702	0.131	–	–
FADEUP	0.456	**0.002**	–	–
FAUP	0.468	**0.002**	–	–
FDUP	0.560	**0.011**	–	–
FPCEUP	0.723	0.169	–	–-

A significance level (*p*) of 0.05 was considered (bolded values)

Ref., reference

## Discussion

The opt-out system raises concerns about exercising individual autonomy, with different points of view existing in the literature. To this end, this study was conducted to evaluate whether young Portuguese people are aware of their country’s presumed post-mortem organ donation law and how it works. Furthermore, we aimed to identify the factors that determine a positive attitude toward post-mortem organ donation.

The results of this study suggest that the majority of students were unaware of current Portuguese legislation. While only 36% of students directly admitted that they did not know about the presumed post-mortem organ donation law, 60% did not recognize that all national citizens as well as stateless persons and foreigners living in Portugal who have not reported their non-donor status to the Ministry of Health are considered potential post-mortem donors. Therefore, we can conclude that more than 36% of the participants, in truth about 60%, were unaware of the legislation. Medical and nursing students were most likely to be aware of the law. In 2009, a Special Eurobarometer survey indicated that only 28% of individuals residing in Europe were aware of the organ donation and transplantation laws that governed their country [18]. Also, health science students were more likely to be knowledgeable about the post-mortem organ donation system than other students, which was also observed by Molina-Pérez et al. [18].

Knowledge of the procedures available to express an individual’s preferences regarding post-mortem organ donation is reported to be lower in opt-out countries than in opt-in countries, as countries with opt-in systems have more campaigns to motivate people to donate [18]. These findings are consistent with our own, as only 7% of all students stated that they knew how to register themselves as non-donors. Furthermore, it was found that 2% of all students were registered as non-donors. This percentage is similar to the results reported by Resende, who found that only 1% of participants had registered themselves as non-donors in RENNDA.

Studies conducted in opt-out countries demonstrate lower favorable attitudes toward presumed organ donation systems [15, 16, 18, 33]. Indeed, only half of all students agreed with the current Portuguese law. These results are similar to those of another Portuguese study [32] in which just 68% of those questioned agreed with the legislation. Nevertheless, medical students showed the most agreement with the law. This positive attitude may be correlated with having a higher degree of information about post-mortem organ donation [18, 33]. On the other hand, law students were the group that was most likely to be against post-mortem organ donation. The main reason given for this negative attitude was the insufficient availability of reliable information about the post-mortem donation system for consent to be presumed. Contrarily, Rydzewska et al. [2] reported that law students have a positive attitude toward the opt-out system. One possible explanation may be that these individuals were more informed about the current policy.

A higher percentage (62%) of all students considered the information to be insufficient. Indeed, the percentage of students who were unaware of the legislation was proportional to the percentage that classified the available information as being insufficient, confusing and inexistent. Thus, we can consider these answers to be coherent, which allows us to exclude the hypothesis of an informed and enlightened population. Furthermore, these 62% are much higher than the percentage found in Resende’s study [32], where 39% of participants considered the available information to be insufficient. This suggests that information about the presumed post-mortem organ donation law has not been effectively disclosed.

Participants who were informed about post-mortem organ donation had sourced their information mainly from social media and the internet. These results were similar to those of other studies, in which the main sources of information mentioned were audio-visual resources [1, 10, 13, 17, 32]. About 22% of students mentioned family as their primary resource, and those students were observed to show a more favorable attitude toward post-mortem organ donation. A review of the literature revealed other studies presenting a positive association between discussing post-mortem organ donation with family and having a favorable attitude toward post-mortem organ donation [10–12, 14, 33, 34]. Furthermore, Siebelink et al. [35] concluded that a child’s education on organ donation is an important factor in fostering family discussion on this topic. Indeed, in recent decades, family systems have changed, making communication between family members more open to different topics [36]. Also, the parent–child relationship has become closer, and these relationships play an important role in the moral lives of young people [36]. Public campaigns also represent an important source of information; in fact, in Sweden, after two information campaigns, young participants’ awareness regarding organ donation and transplantation increased from 18% in 2001 to 40% in 2005 [18]. However, in our study, only 6% had been informed by these means. This result is similar to that found in Resende’s study [32], where only 7% of the population had been informed by public campaigns in 2008. According to the international literature, society and governments should invest in educating the public, through either public campaigns or media resources such as television and the internet [1–4, 13, 16, 18]. Furthermore, 10% of all participants in our study got their informed from health professionals, and despite this being a small percentage, it still represents a relevant portion of the study population. Because health professionals are presented as one of the main sources of information to the public, they must be sensitive to the importance of post-mortem organ donation while maintaining a positive attitude toward the opt-out system [1, 9, 33].

Only 3% of students who came from Portuguese secondary schools said that the subject of post-mortem organ donation had been discussed during classes. However, approximately 21% of participants who came from non-PALOP countries had talked about post-mortem organ donation in secondary school. This suggests that this subject is more frequently discussed in non-PALOP countries, especially Brazil. This could be because opt-in countries have a greater need to motivate people to donate, and schools represent an important source of information for much of the population [18], such that almost all participants agreed that this subject should be discussed in school. Indeed, three studies were found to recommend the introduction of educational programs and/or campaigns about organ donation in school [2, 4, 17].

Women were shown to be more in favor of the legislation, which may be due to differences in altruism, as seen in an earlier study showing that women are more altruistic than men [16]. Other studies report similar results [9, 11, 12].

Almost all students correctly understood the concept of brain death. However, only 64% were aware of the possibility of organ donation from a brain-dead person. Regarding this topic, different results were found in the international literature. For example, Al Bshabshe et al.’s study [17] found that only 47% of a sample of Saudi Arabian students was aware of the concept of brain death, while only 21% of students were aware of the possibility of donation from a brain-dead person. Meanwhile, Janahia et al.’s study [5] in the United Arab Emirates found that over 88% of the population understood the concept of brain death and 76% were aware that patients who had experienced irreversible brain death and were maintained on life support could be considered potential post-mortem organ donors; the latter results were more consistent with our own. Of the students who were undecided about the possibility of organ donation from a brain-dead person, only 37% agreed with the current legislation. Some authors have concluded that awareness of the concept of brain death is associated with a more favourable attitude toward post-mortem organ donation [10, 12, 14].

In terms of the influence of religion on attitudes toward post-mortem organ donation, the results presented in the international literature are not always consistent. Some studies have shown that the most favorable attitudes were found among atheists and/or agnostics compared to Catholics and members of other religions [12, 13, 34]. On the other hand, a few studies found that Catholics were more likely to be in favor of post-mortem organ donation [20, 33, 34]. Furthermore, one study showed no correlation, but concluded that having knowledge of the Catholic Church’s positive stance on post-mortem organ donation was associated with a more favorable attitude compared to when people were unaware of the Catholic Church’s position on this subject [11]. In our study, no correlation was found between religion and attitude toward organ donation, which was similar to the results of two other studies [10, 14].

Twenty-seven years have passed since the law was changed and Portugal became an opt-out system, so it would be expected that the majority of the population should be aware of the legislation. However, the results of our study have indicated the opposite, perhaps due to a lack of dissemination of information about this system. Although the government, according to current law, has a duty to inform the population through public campaigns, these campaigns have been scarce and usually take place in primary health centers or public hospitals, which may restrict the public’s access to this information, given that some Portuguese citizens choose to use private health services as an alternative to the National Health System [37]. This lack of information may allow us to reflect upon another important area related to respect for individual autonomy. To maintain respect for individual autonomy in an opt-out system, the population must be informed about the law, because if this does not happen, organ recovery may occur after the death of those who never truly consented. In this regard, Beauchamp and Childress [28] state that opt-out systems are ethically justifiable if the government requires vigorous efforts be made to ensure public understanding of the options they face as individuals, including how to opt out. If governments do not ensure that their populations are informed about the law on post-mortem organ donation, they are not respecting the individual autonomy of their citizens. In fact, they are camouflaging them through presumed consents and may indeed be acting against the will of the individual while alive. The Portuguese people need to be informed and enlightened in a regular and systematic way about all the steps they must follow to freely express their willingness to be organ donors or not. This is essential for the system of presumed donation of organs established in Portugal to reflect the conscious choice of each citizen, otherwise the lack of information could undermine the legitimacy of presumed donated in an opt-out system. Autonomous persons are aware, informed and free in their decision-making.

Given this, and considering that the Portuguese population is poorly informed, it is important that the government outline strategies to amend this knowledge gap. The inclusion of this topic in schools, such as in science programs or even in citizenship education programs, is probably the best way to educate younger generations on this topic. An informed young person will be an informed adult citizen. On the other hand, social media (television, radio, internet) and public campaigns can be used to inform adults and older generations.

Our study is limited by the use of a non-validated questionnaire, which could lead to incorrect interpretation and generalization of the results. Another limitation related to the questionnaire is the response bias, which stems from how the questions were designed and the knowledge and understanding of the participants. This bias makes it difficult to estimate how well the questions and answers represent the related topic. Additionally, this study was conducted in only six faculties of the University of Porto and had a small sample size (n = 841); therefore, any generalization of the results must be done with care.

The strengths of this study were mostly related to the participation of our sample; the questionnaire’s completion rate was 89% and no participants refused to participate in the study. Students had no difficulty understanding or completing the survey in the proposed time, which suggests that the questions were clear and succinct. Another positive point was that the sample was heterogeneous and included individuals from countries whose secondary school education systems and education programs are different from those of Portugal. This study also contributed, on a small scale, to raising awareness among students, as some of them stated that they had become aware of the issue tanks to the survey.

In Portugal, findings relating to citizens’ awareness of the post-mortem organ donation law are scarce, with only one study being found [32]. Therefore, it would be interesting to extend this study beyond the current population of 18-year-old Portuguese students, not only to acquire a larger sample of participants but also to obtain a sample with individuals of different ages, marital statuses, economic strata, and levels of education.

## Conclusions

There is a significant lack of knowledge among young Portuguese people regarding aspects of the presumed post-mortem organ donation law and how it works. Indeed, a high percentage of students considered the available information to be insufficient and almost all agreed that this subject should be discussed in secondary school. Furthermore, only half of all participants agreed with the current opt-out system.

Being of the female sex, having family as a primary source of information, and being aware of presumed post-mortem organ donation law are the strongest independent factors in determining a positive attitude toward post-mortem organ donation.

According to international literature and the results of our study, the importance of increased public awareness of presumed post-mortem organ donation law is clear, as this is associated with greater agreement with the current law. The population can be informed through public campaigns and formal education, with program curricula including the concepts of brain death and post-mortem organ donation law. The literature also demonstrates the importance of educating individuals so that they understand the advantages of post-mortem organ donation, thereby improving the number of available organs and, consequently, decreasing the organ shortage and associated waiting list mortality. Furthermore, our results and the literature show that having family as the primary source of information is an important factor in determining one’s attitudes toward post-mortem organ donation. For that reason, dialogue about this subject within family circles should be encouraged as a means of demystifying death.

Finally, taking the perspective of many other authors, if the right to the freedom of self-determination of all human beings is to be respected, it is essential that these human beings be informed, which should motivate governments to promote public campaigns and other measures to inform the populace.

## Data Availability

The datasets generated and/or analyzed in this study are not publicly available due to ethical reasons, but are available from the corresponding author upon reasonable request.

## Abbreviations

RENNDA: National Register of Non-Donors
IPST: Portuguese Institute of Blood and Transplantation
CVA: Cerebrovascular accident
FMUP: Faculty of Medicine of the University of Porto
ESEP: Faculty of Nursing of the University of Porto
FPCEUP: Faculty of Psychology and Education Sciences of the University of Porto
FDUP: Faculty of Law of the University of Porto
FAUP: Faculty of Architecture of the University of Porto
FADEUP: Faculty of Sports of the University of Porto
PALOP countries: African countries where the official spoken language is Portuguese
non-PALOP countries: Non-African countries where Portuguese is the official spoken language
